# Deep learning enhances the prediction of HLA class I-presented CD8^+^ T cell epitopes in foreign pathogens

**DOI:** 10.1038/s42256-024-00971-y

**Published:** 2025-01-28

**Authors:** Jeremy Wohlwend, Anusha Nathan, Nitan Shalon, Charles R. Crain, Rhoda Tano-Menka, Benjamin Goldberg, Emma Richards, Gaurav D. Gaiha, Regina Barzilay

**Affiliations:** 1 Department of Electrical Engineering and Computer Science, Massachusetts Institute of Technology, Cambridge, MA USA; 2 Jameel Clinic, Massachusetts Institute of Technology, Cambridge, MA USA; 3 Ragon Institute of Mass General, MIT and Harvard, Cambridge, MA USA; 4 Program in Health Sciences and Technology, Harvard Medical School and Massachusetts Institute of Technology, Boston, MA USA; 5 Division of Gastroenterology, Massachusetts General Hospital, Boston, MA USA

**Keywords:** MHC class I, Antigen presentation

## Abstract

Accurate in silico determination of CD8^+^ T cell epitopes would greatly enhance T cell-based vaccine development, but current prediction models are not reliably successful. Here, motivated by recent successes applying machine learning to complex biology, we curated a dataset of 651,237 unique human leukocyte antigen class I (HLA-I) ligands and developed MUNIS, a deep learning model that identifies peptides presented by HLA-I alleles. MUNIS shows improved performance compared with existing models in predicting peptide presentation and CD8^+^ T cell epitope immunodominance hierarchies. Moreover, application of MUNIS to proteins from Epstein–Barr virus led to successful identification of both established and novel HLA-I epitopes which were experimentally validated by in vitro HLA-I-peptide stability and T cell immunogenicity assays. MUNIS performs comparably to an experimental stability assay in terms of immunogenicity prediction, suggesting that deep learning can reduce experimental burden and accelerate identification of CD8^+^ T cell epitopes for rapid T cell vaccine development.

## Abstract (web summary)

Accurate prediction of immunogenic CD8^+^ T cell epitopes would greatly accelerate T cell vaccine development. A new deep learning model, MUNIS, can rapidly identify HLA-binding, immunogenic and immunodominant peptides in foreign pathogens.

## Main

Cytotoxic CD8^+^ T cells have been shown to limit disease severity and provide protection against viral infections such as human immunodeficiency virus (HIV)^1,2^ and severe acute respiratory syndrome coronavirus 2 (SARS-CoV-2)^3–6^ by recognizing short viral peptides bound to human leukocyte antigen class I (HLA-I) molecules. While T cell-based vaccines hold great promise for foreign pathogens^2,4,7–10^, rapidly identifying immunogenic epitopes across viral proteomes is challenging owing to the extensive degree of HLA polymorphism in the population and the experimental burden required to validate HLA-I binding and CD8^+^ T cell reactivity. Given that only a small fraction of pathogen-derived peptides elicit an in vivo CD8^+^ T cell response^11,12^, computational algorithms that can rapidly identify immunogenic epitopes within viral proteomes for a broad range of HLA-I alleles would greatly accelerate T cell-based vaccine development.

Although numerous computational methods exist to predict CD8^+^ T cell epitopes^13–16^, their accuracy varies substantially across HLA-I alleles^17^. Moreover, the extent to which these models can identify immunogenic epitopes is not well understood, in part owing to the lack of unbiased evaluation datasets. Peptides frequently tested for immunogenicity are often conditionally selected using existing binding predictors^13–16^. Although several tools can define the characteristics of immunogenic cancer neoepitopes^18–23^, they are yet to show strong generalizability to foreign pathogens. Thus, given the remarkable success of deep learning to improve model generalization across several areas of biology, such as protein structure prediction^24^ and CD4^+^ T cell epitope presentation^25,26^, we reasoned that it could also enhance the prediction of presented and immunogenic CD8^+^ T cell epitopes.

We therefore developed MUNIS: a deep learning-based predictor of HLA-I epitopes that utilizes a bimodal architecture to jointly model both HLA-I–peptide binding and antigen processing by leveraging a well-curated and expanded training set of 651,237 unique HLA-I ligands across 205 HLA-I alleles. We validated MUNIS in silico on immunopeptidomic data and achieved superior performance compared with existing HLA-I epitope predictors. Distinct from previous work^13–16^, we evaluated immunogenicity prediction using several unbiased immunopeptidomic benchmarks. We subsequently validated MUNIS experimentally using an in vitro HLA-I–peptide stability assay^27^ and T cell immunogenicity assays on predicted peptides from Epstein–Barr virus (EBV). We explicitly omitted all peptides in EBV from the model training set to mimic prediction of a novel virus. Importantly, MUNIS identified both established and new CD8^+^ T cell epitopes in EBV that elicited effector and memory CD8^+^ T cell responses. Moreover, MUNIS was a comparable immunogenicity predictor to an HLA-I–peptide stability assay^27^, which defined epitopes for a SARS-CoV-2 T cell vaccine^28^, illustrating the power of deep learning to reduce experimental burden and rapidly accelerate T cell vaccine development.

## Deep learning model development for CD8^+^ T cell epitope identification

**Fig. 1:**
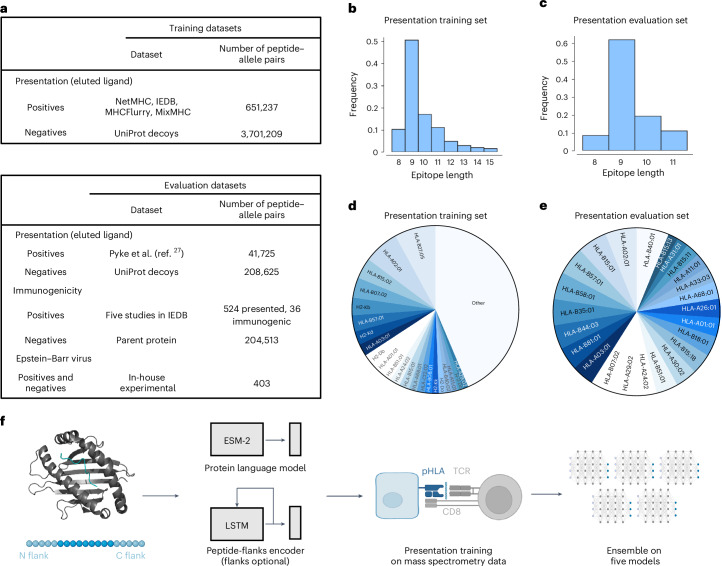
Characteristics of the deep learning model and the training and evaluation datasets for prediction of HLA-I epitopes. **a**, Datasets used for training and evaluation were curated by combining data from several previous studies as well as a recent download of the IEDB. Eluted ligand data were used as positives and randomly sampled decoys from Swiss-Prot^26^ served as negatives. For evaluation, data from an immunopeptidomic study involving 24 monoallelic cell lines were used^27^. To evaluate immunogenicity, five studies^28–30^ that measure the immunogenicity of influenza epitopes identified via mass spectrometry were used. **b**–**e**, Peptide length distribution of the HLA-I binders (**b**,**c**) and pie chart of the proportion of epitopes per HLA-I allele (**d**,**e**) in the presentation training (**b**,**d**) and evaluation (**c**,**e**) datasets. All alleles present in the dataset with a frequency <1% are denoted as ‘other’. **f**, The binding module takes as input the amino acid sequences of the major histocompatibility complex and peptide in the form: [cls] mhc [sep] pep [eos], where [cls], [sep] and [eos] are special tokens that separate the two sequences. This new sequence is fed to the Evolutionary Scale Modeling-2 (ESM-2) Transformer protein language model, and the vector representation for the [cls] token is used to represent the complex. The ligand elution module combines the binding vector with a long short-term memory (LSTM) recurrent neural network encoding of the peptide that includes its left and right flanks in the parent protein of origin. The model can be used when trained with or without flanking residues. These combined features are then concatenated and used to compute a ligand presentation score. The model is first trained on the ligand presentation task. Then, the model is trained with five different random seeds and their scores are averaged to create an ensemble score. pHLA: peptide-human leukocyte antigen complex; TCR: T cell receptor. Panel **f** created with BioRender.com.

To develop a robust model of immunogenic CD8^+^ T cell epitope prediction, we constructed a training dataset composed of mass spectrometry data from immunopeptidomic experiments used for the MixMHCpred2.2^16^, NetMHCpan4.1^13^ and MHCflurry2.0^14^ training sets and data obtained from the Immune Epitope Database (IEDB: iedb.org)^29^. We supplemented these HLA-I-binding peptides with randomly sampled decoy peptides (non-binders) from Swiss-Prot^30^ at a ratio of 1:5 HLA-I binders to non-binders. Importantly, all epitopes used in model evaluation were removed from training sets, regardless of HLA-I restriction. This is a far stricter approach to data filtering than previous methodologies where only matched HLA-I–peptide pairs were removed from the training set, resulting in substantial overlap between test set epitopes and training sets. We show the sources of these datasets (Fig. 1a), the peptide length distributions (Fig. 1b,c) and the frequencies at which peptides bind HLA-I molecules (Fig. 1d,e).

The architecture of the model is composed of two submodules (Fig. 1f). First, the binding module takes as input the sequences of the HLA-I molecule and peptide and learns a numerical vector representation, which we refer to as binding features, to produce the final prediction score. Our second module augments the binding features with a signal relevant to antigen processing. This is accomplished by encoding the peptide and five N-terminal and C-terminal flanking residues from its parent protein of origin, similar to MHCflurry2.0^14^. Using this approach, we train five models and combine them in an ensemble by taking the average score on any given input. The score of the model for HLA-I–peptide pairs ranges between 0 and 1 and indicates the probability of a peptide binding that particular HLA-I allele.

Previous work has frequently relied on area under the receiver operating characteristic curve (ROC-AUC) and precision (that is, positive predictive value) as evaluation metrics. However, given the abundance of negatives, it is possible that for a given threshold, the proportion of positives is dominated by false positives even with a low false-positive rate. Thus, we primarily use the area under the precision–recall curve, known as average precision score, to describe the model performance. This score ranges from 0 to 1, with a random predictor having a value equal to the percentage of positives in the data. For datasets of sufficient size, we also show the ROC-AUC.

## MUNIS outperforms existing predictors in classifying HLA-I binders

**Fig. 2:**
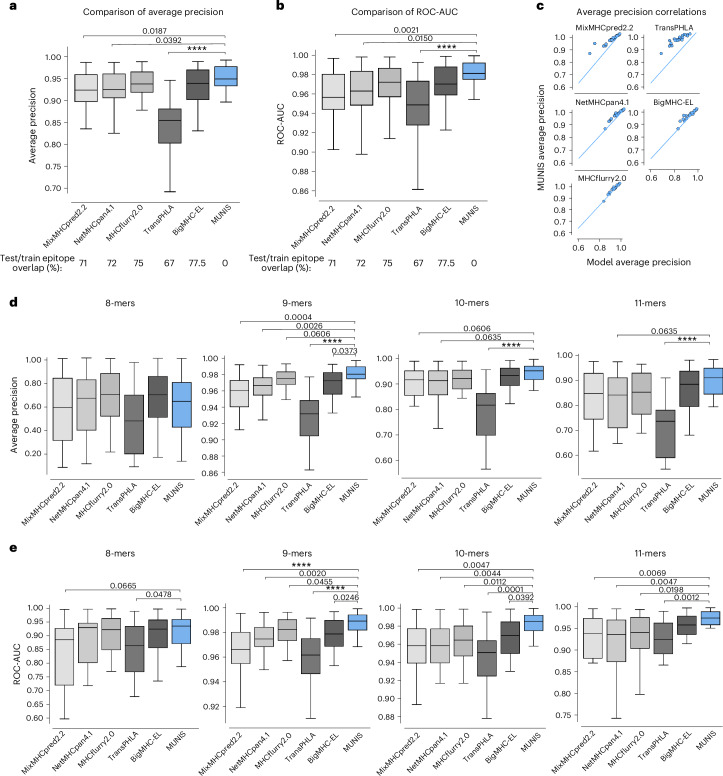
MUNIS outperforms existing predictors in classifying HLA-I binders across 8–11mers. **a**,**b**, Average precision (**a**) and ROC-AUC (**b**) of MUNIS and current state-of-the-art tools MixMHCpred 2.2, NetMHCpan 4.1, MHCflurry 2.0, TransPHLA and BigMHC on predicting eluted ligands (binders) from mass spectrometry experiments from Pyke et al.^27^ against decoy peptides (non-binders), *n* = 24 HLA-I alleles. Percentages of overlap with the training datasets of each tool across all epitopes in the presentation benchmark are shown below the plots. **c**, Per-allele pairwise comparisons of MUNIS and other predictors in classifying HLA-I binders. Each point is the model performance on one allele. **d**,**e**, Average precision (**d**) and ROC-AUC (**e**) of all predictors on classifying binders versus non-binders binned by epitope length, *n* = 24 HLA-I alleles. *P* values for pairwise comparisons between MUNIS and each predictor were calculated using the two-sided Wilcoxon rank sums test (not shown if *P* > 0.1; ****P < 1 × 10^-4^). Box plots are presented with medians as centre lines, 25th and 75th percentiles as lower and upper quartiles, and 1.5 times the interquartile range from the quartiles as whiskers (outliers not shown).

We first evaluated the presentation model on a published immunopeptidomic dataset^31^ that contains 41,725 positive HLA-I–peptide pairs and 208,625 randomly sampled decoys across 24 HLA-I alleles. We calculated the average precision scores and ROC-AUC of classifying binders and non-binders on a per-allele basis and compared our scores with the existing tools MixMHCpred2.2, NetMHCpan4.1, MHCflurry2.0, TransPHLA^32^ and BigMHC^33^, which predicts both presented peptides (BigMHC-EL) and immunogenic epitopes (BigMHC-IM). On this dataset, our model achieves a median average precision of 0.952, which corresponds to a 21% reduction in error compared with existing tools, with MixMHCpred2.2 scoring 0.924, NetMHCpan4.1 scoring 0.925, MHCflurry2.0 scoring 0.938, TransPHLA scoring 0.854 and BigMHC scoring 0.939 (Fig. 2a). The median ROC-AUC of MUNIS is 0.980, which corresponds to a 31% reduction in error compared with MHCflurry2.0 at 0.971, NetMHCpan4.1 at 0.962, MixMHCpred2.2 at 0.956, TransPHLA at 0.948 and BigMHC at 0.969 (Fig. 2b). Importantly, over 65% of positive epitopes in the evaluation dataset are also present in the training sets of existing predictors, excluding MUNIS where we ensure a 0% overlap. Despite this overlap, MUNIS still outperforms these prediction algorithms on 22/24 HLA-I alleles tested in average precision (Fig. 2c).

Evaluation of these various predictors suggests that differences in model performance could be due to each model’s ability to encode peptide length^16^. We therefore evaluated model performance across HLA-I alleles stratified by peptide length (Fig. 2d,e). This revealed that MUNIS outperforms for 9-mer, 10-mer and 11-mer peptides, indicating that encoding of peptide lengths is unlikely to be a key discriminator of model predictive capability.

**Extended Data Fig. 1:**
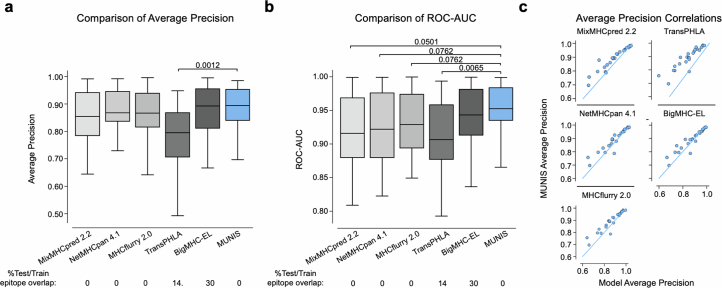
MUNIS outperforms state-of-the-art predictors in classifying HLA-I binders using a test set with no overlap with the training set. **(a)** Average precision and **(b)** ROC-AUC of all predictors on classifying binders versus non-binders using the subset of the data with no epitope overlap with any of the tools’ training datasets (“cleaned evaluation set”), n = 24 alleles. Box plots are presented with medians as center lines, 25^th^ and 75^th^ percentiles as lower and upper quartiles, and 1.5 x interquartile range from the quartiles as whiskers (outliers not shown). **(c)** Per allele pairwise comparisons of MUNIS and other predictors in classifying HLA-I binders using the cleaned evaluation set. Each point is model performance on one allele. P values for pairwise comparisons between MUNIS and each predictor were calculated using the two-sided Wilcoxon rank sums test (not shown if p > 0.1).

To further evaluate predictive capabilities, we compared MUNIS with existing predictors using the identical evaluation dataset as above but with most peptides included in the tool training sets removed. In this setting, we observed a wider gap in performance for several HLA-I alleles, with MUNIS achieving a median average precision score of 0.894, MixMHCpred2.2 scoring 0.854, NetMHCpan4.1 scoring 0.868, MHCflurry2.0 scoring 0.867, TransPHLA scoring 0.795 and BigMHC scoring 0.891 (Extended Data Fig. 1a). MUNIS outperforms existing tools on 18/24 HLA-I alleles tested in average precision and 21/24 in ROC-AUC (Extended Data Fig. 1b), and a larger performance gap was observed for several HLA-I alleles on this cleaned evaluation set against all tools, except for BigMHC which retains 30% overlap with positive epitopes in the clean test set and TransPHLA which retains 14% overlap (Extended Data Fig. 1c). These data reveal that MUNIS can identify presented peptides from mass spectrometry data with greater accuracy than existing tools across several source proteins, HLA-I alleles and peptide lengths.

## MUNIS predicts fewer false positives by using canonical HLA-I motifs

**Fig. 3:**
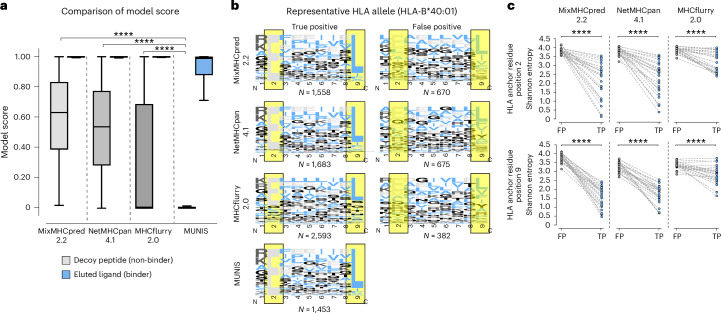
Motif analysis of misclassified binders reveals inconsistent reliance of existing models on canonical HLA-I-binding motifs. **a**, Box plots of model score for eluted ligands (binders) from mass spectrometry experiments from Pyke et al.^27^ and decoy peptides (non-binders) for each predictor (41,724 binders and 208,609 non-binders). Box plots are presented with medians as centre lines, 25th and 75th percentiles as lower and upper quartiles, and 1.5 times the interquartile range from the quartiles as whiskers (outliers not shown). **b**, Binding motifs for 9-mers for all correctly classified binders (true positives) and misclassified non-binders (false positives) by each tool for representative allele HLA-B*40:01. HLA anchor residues are highlighted in yellow. Binding motifs are not shown for MUNIS false positives as there were fewer than 25 incorrectly labelled binders per allele. Model scores >0.90 were used as cut-offs for true positives and false negatives. **c**, Shannon entropy at HLA anchor residues (positions two and nine in a 9-mer) for true-positive (TP) and false-positive (FP) HLA-I binders predicted by each tool. Each point represents the Shannon entropy at a particular anchor residue for peptides that are false and true predicted binders for one HLA allele. *P* values for pairwise comparisons were calculated using the two-sided Wilcoxon rank sums test (****P < 1 × 10^−4^).

Given the improved performance of MUNIS in predicting HLA-I-presented peptides, we evaluated peptide-binding motifs for several alleles. Interestingly, we found that existing tools assign high individual model scores to non-HLA-I-binding peptides and thereby predict an increased number of false positives (Fig. 3a). We therefore evaluated the HLA-I-binding motifs for correctly classified peptides (true positives) versus misclassified peptides (false positives) for all HLA-I alleles in the evaluation set, with HLA-B*40:01 shown as a representative example (Fig. 3b). While all predictors were capable of correctly identifying peptides with canonical binding motifs, MixMHCpred2.2, NetMHCpan4.1 and MHCflurry2.0 also classified peptides with non-canonical anchor residue motifs as binders as well. The binding motifs of the false positives across these three predictors did not have a dominant amino acid at HLA anchor residue positions (Fig. 3b, highlighted in yellow) as observed for true positives. In addition, falsely classified binders have significantly greater entropy at HLA anchor residues compared with true binders (Fig. 3c). In contrast, MUNIS largely avoids classifying peptides without expected HLA anchor residues as positives and, consequently, false positives were extremely low for MUNIS with fewer than 25 misclassified peptides per HLA-I allele.

## MUNIS predicts epitope immunogenicity and immunodominance

Given that only a fraction of HLA-I-presented peptides elicit CD8^+^ T cell responses, we next measured the ability of MUNIS to predict immunogenic epitopes. To avoid biases from existing prediction algorithms in peptide selection, we constructed a test set of peptides using five immunopeptidomic datasets from the influenza viruses A and B that bind to HLA-A*02:01, HLA-A*11:01 and HLA-A*24:02 (524 presented peptides across the five datasets)^34–36^. Each of these datasets comprises a list of HLA-I-presented peptides identified via mass spectrometry that were subsequently evaluated for immunogenicity using interferon-γ (IFNγ) enzyme-linked immunospot (ELISpot) assays, where 36 of the 524 peptides were reported as immunogenic in the IEDB. For each dataset, we ranked positive (that is, immunogenic) peptides against all other peptides in the viral proteome. When compiling the set of negatives, we considered only proteins with at least one immunogenic peptide to prevent confounding by inherent levels of protein immunogenicity.

**Fig. 4:**
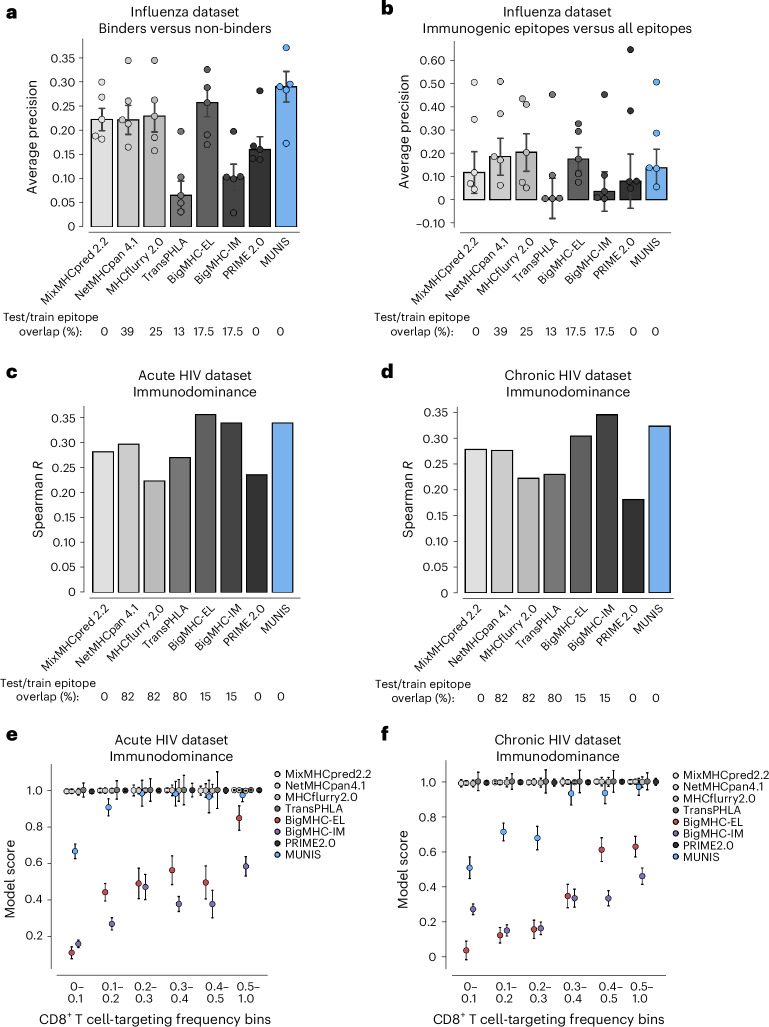
MUNIS outperforms existing tools in predicting epitope immunodominance hierarchies. **a**, Per-dataset performance of MUNIS against existing tools MixMHCpred 2.2, NetMHCpan 4.1, MHCflurry 2.0, TransPHLA, BigMHC and Prime 2.0 on predicting eluted ligands (binders) from five influenza immunopeptidome experiments against ‘decoy’ peptides (non-binders). Positives are all mass spectrometry-eluted ligands and negatives are all other peptides (‘decoys’) in the viral proteome. Only proteins with at least one eluted ligand are considered. **b**, Per-dataset performance when positives are conditioned on immunogenic peptides and negatives contain both the ‘decoys’ and the eluted ligands that were not immunogenic. In **a** and **b**, each point represents performance on one dataset (that is one HLA-I allele). Bar plots show median performance across datasets and error bars show the standard error across the five datasets. Percentages of epitope overlap with the training datasets of each tool across all positive epitopes in the five influenza benchmarks are shown below the plots. No pairwise comparisons between MUNIS and other predictors had a *P* value <0.05. **c**,**d**, Spearman correlation of each model’s score and frequency of response to an epitope across all epitope–allele pairs in acute (**c**) and chronic (**d**) HIV infection. Percentages of epitope overlap with the training datasets of each tool across all epitopes in the HIV benchmark are shown below the plots. **e**,**f**, Median model score ± standard error of the median for epitopes with binned frequencies of responses across all epitope–allele pairs in acute (**e**) and chronic (**f**) HIV infection.

**Extended Data Fig. 2:**
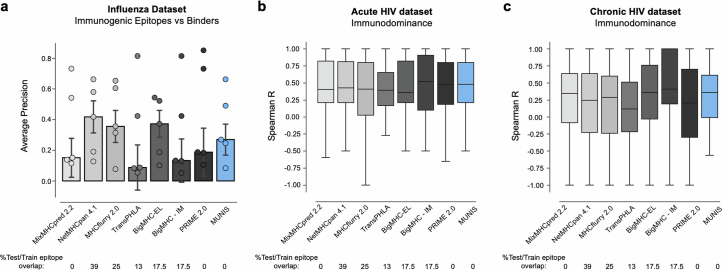
Performance of all algorithms in predicting epitope immunogenicity conditioned on HLA-I-peptide binders and immunodominance stratified by HLA-I allele. **(a)** Per-dataset performance of our model, MUNIS, against existing tools MixMHCpred2.2, NetMHCpan4.1, MHCFlurry2.0, TransPHLA, BigMHC and PRIME2.0 in predicting eluted ligands from the five influenza immunopeptidomic experiments against decoy peptides. Positives are all immunogenic epitopes and negatives are all eluted ligands (i.e binders) in the viral proteome. Error bars show the standard error across the five datasets. Bar plots show median values. Spearman correlation of each model’s score and the frequency of response across all epitope-allele pairs in **(b)** acute (n = 33 alleles) and **(c)** chronic (n = 33 alleles) HIV. Bar plots show medians and error bars show the standard error across alleles.

We benchmarked MUNIS against immunogenicity predictors PRIME2.0^14^ and BigMHC_IM and the HLA presentation prediction tools (MixMHCpred2.2, NetMHCpan4.1, MHCflurry2.0, TransPHLA and BigMHC_EL). PRIME2.0 was benchmarked on immunogenicity test sets by running each allele–peptide pair independently using ‘%Rank_bestAllele’ as a readout of prediction strength. We found that MUNIS outperforms all other prediction algorithms in identifying presented epitopes across the 5 datasets, with a median average precision of 0.289, which is a 26% relative improvement in performance compared with the next-best tool (MHCflurry2.0; Fig. 4a). However, all tools showed a similar predictive capability across HLA-I alleles when predicting immunogenic epitopes against decoy peptides (Fig. 4b). Of note, the tools with higher median average precision scores than MUNIS were the tools with substantial overlap between evaluation and training sets. To further understand this result, we measured performance with only non-immunogenic HLA-I binders as negatives (Extended Data Fig. 2a). This resulted in increased average precision over several alleles, indicating the tendency of models to rank presented, immunogenic epitopes higher than presented, non-immunogenic ones. These results highlight the intricate relationship between an increased likelihood of presentation and downstream T cell recognition and also underscore the unresolved gap to achieve high accuracy for immunogenicity when filtered on known HLA-I binders.

While notable differences in immunogenicity prediction for individual epitopes were not observed, we explored whether MUNIS could predict immunodominance hierarchies, as effective T cell vaccines would ideally elicit immune responses across many individuals. We therefore leveraged T cell response data for HIV given known immunodominance hierarchies for multiple HLA-I alleles^37^. Specifically, we used a dataset where 119 HIV epitopes were tested for CD8^+^ T cell responses in 527 individuals with HIV split across acute and chronic infection groups^18,24^, with each peptide eliciting a CD8^+^ T cell response in up to 81% of corresponding individuals with HLA-I^+^.

We evaluated our model by computing the Spearman rank correlation coefficient between the MUNIS-predicted score and response frequency for each epitope. MUNIS had a Spearman correlation coefficient of 0.35 compared with 0.34 and 0.295 for the next-best-performing tools (BigMHC_IM and NetMHCpan4.1) for epitopes targeted in the acutely infected HIV^+^ subgroup (Fig. 4c) and 0.33 compared with 0.34 and 0.28 (BigMHC and MixMHCpred 2.2) for epitopes targeted in the chronically infected HIV^+^ subgroup (Fig. 4d). Of note, three tools used for comparison (NetMHCpan4.1, MHCflurry2.0, BigMHC) were enriched for HIV epitopes in their training dataset, whereas these were excluded from the MUNIS training set. We also stratified performance on a per-allele basis and found that MUNIS outperforms in both the acute (Extended Data Fig. 2b) and chronic (Extended Data Fig. 2c) subgroups. Overall, these data demonstrate that MUNIS is competitive in identifying immunodominant CD8^+^ T cell epitopes despite not having been explicitly trained for this task.

Because of the numerous false-positive binders predicted by other tools, we hypothesized that the superior capability of MUNIS to recapitulate immunodominance hierarchies could be due to prediction scores that reflect the targeting frequency of epitopes. When we binned epitopes by targeting frequency, we again observed that more conservative prediction scores from MUNIS discriminate between subdominant and immunodominant epitopes in HIV (Fig. 4e,f), with BigMHC being the only other tool to share this property. Collectively, these data demonstrate that MUNIS can identify epitopes with higher likelihoods of eliciting CD8^+^ T cell responses broadly across individuals.

**Extended Data Fig. 3:**
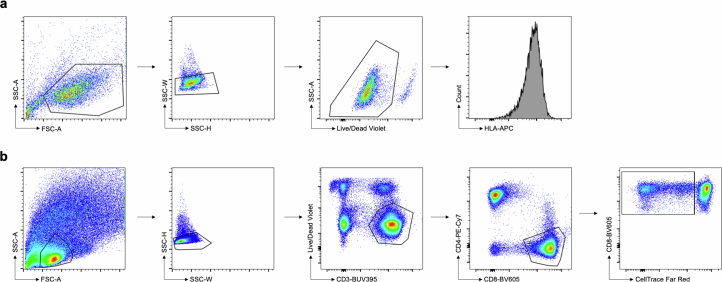
Flow cytometry gating strategies for HLA class I-peptide stability and proliferation assays. **(a)** TAP-deficient monoallelic HLA class I-expressing 721.221 cells were stained with viability dye and pan-HLA antibody W6/32. **(b)** Representative gating strategy for identification of proliferating CD3^+^ CD8^+^ CellTrace Far Red^l^° T cells in response to peptides of interest.

To deconvolute which features contribute to enhanced model performance, we performed an extensive ablation analysis with competing models (Extended Data Fig. 3). We selectively ablated individual features to query the impact of the pretrained Evolutionary Scale Modeling-2 (ESM-2) Transformer protein language model, size of the language model, flanking residues, negative sampling logic and model score versus percentage rank score, which attempts to normalize model scores across HLA-I alleles (Extended Data Fig. 3b). A larger ESM-2 model (35 million parameters) improved performance slightly over a smaller model (8 million parameters), with an average precision of 0.959 compared with 0.953, respectively. Pretraining the model also improved the prediction of HLA-I–peptide presentation from an average precision of 0.946 to 0.953. Introducing flanking residues enhanced HLA-I–peptide presentation prediction from an average precision of 0.947 to 0.953. Interestingly, many of these features (namely, pretraining and incorporating flanking residues) slightly reduced the average precision on immunogenicity prediction. With regards to immunodominance, where the results are not stratified by allele, we compared the performance of MUNIS using the native score and the percentage rank for any given peptide and found that both methods perform similarly on the HIV acute and chronic immunodominance datasets. Finally, we observed that sampling decoys from Swiss-Prot was generally equivalent to sampling solely from the human proteome.

## MUNIS discriminates between HLA-I-binding and non-binding EBV peptides

**Fig. 5:**
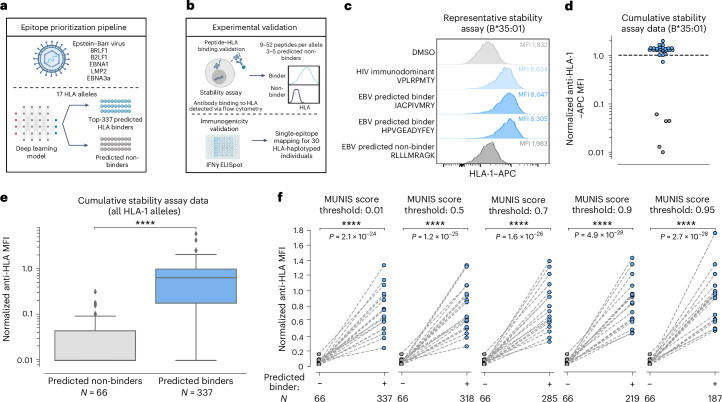
Experimental HLA-I–peptide stability assay confirms the ability of MUNIS to discriminate between binding and non-binding peptides within EBV. **a**, Schematic showing the epitope prioritization pipeline for experimental validation. The top-337 ranked peptides from the BRLF1, B2LF1, EBNA1, LMP2 and EBNA3a proteins from EBV predicted to bind 1 of 17 different HLA-I alleles were chosen for downstream analysis. **b**, Schematic showing experimental validation of MUNIS performance on EBV epitope prediction. Stability assays on HLA-I–peptide pairs were performed using TAP-deficient monoallelic HLA-I cell lines to identify peptides that bind and are presented by HLA-I molecules. IFNγ ELISpot assays were performed on each peptide predicted to bind an HLA molecule presented by 30 HLA-haplotyped individuals to identify immunogenic peptides (data shown in Fig. 6). **c**, Representative data of the relative stabilization of HLA-B*35:01 by two EBV peptides predicted to bind the allele. The MFI for the DMSO negative control shown in light grey, the B*3501-specific HIV immunodominant peptide in light blue, the two predicted binders from the EBV proteome in blue and a predicted non-binder from the EBV proteome in dark grey. The higher the MFI, the greater stabilized the allele by a given peptide. **d**, Summary data for all predicted binders and non-binders for HLA-B*35:01. All MFIs were normalized to the HIV immunodominant peptide for the given HLA-I allele as denoted by the dashed line. Blue circles are predicted binders and grey circles are predicted non-binders. **e**, Summary data for all 17 HLA-I alleles evaluated for the 337 predicted peptides. Box plots are presented with medians as centre lines, 25th and 75th percentiles as lower and upper quartiles, and 1.5 times the interquartile range from the quartiles as whiskers (outliers not shown). **f**, Normalized anti-HLA MFI for binders versus non-binders conditioned on predicted binders with a MUNIS score greater than or equal to the given threshold score. Each point represents the median normalized anti-HLA MFI across all peptides predicted to bind or not bind a particular HLA-I allele (*n* = 17 HLA-I alleles). *P* values for pairwise comparisons between predicted binders and non-binders were calculated using the two-sided Wilcoxon rank sums test. Panels **a** and **b** created with BioRender.com.

To evaluate the practical utility of MUNIS, we experimentally assessed predictions of epitope binding and immunogenicity within the EBV proteome for several HLA-I alleles, given its relevance to immunocompromised populations^38^, linkage to multiple sclerosis^39,40^ and >90% prevalence in the human population^41^. For this evaluation, we made EBV a de novo virus by excluding epitopes from the EBV proteome from the MUNIS training set. We first scanned all 8–15mers from 5 immunogenic EBV proteins (BRLF1, B2LF1, EBNA1, LMP2 and EBNA3a) and selected 337 peptides predicted to bind 17 HLA-I alleles using an earlier iteration of MUNIS. All of these peptides had scores >0.01 upon model finalization (Fig. 5a). Predicted binders for one allele were used as predicted non-binders for another allele if the MUNIS score was less than 0.01, contributing to a robust immunogenicity evaluation set. Each peptide was evaluated for HLA-I binding using an established HLA-I–peptide stability assay^27^ and subsequently tested for T cell immunogenicity by IFNγ ELISpot assays in peripheral blood mononuclear cells (PBMCs) obtained from HLA-haplotyped human participants (Fig. 5b).

**Extended Data Fig. 4:**
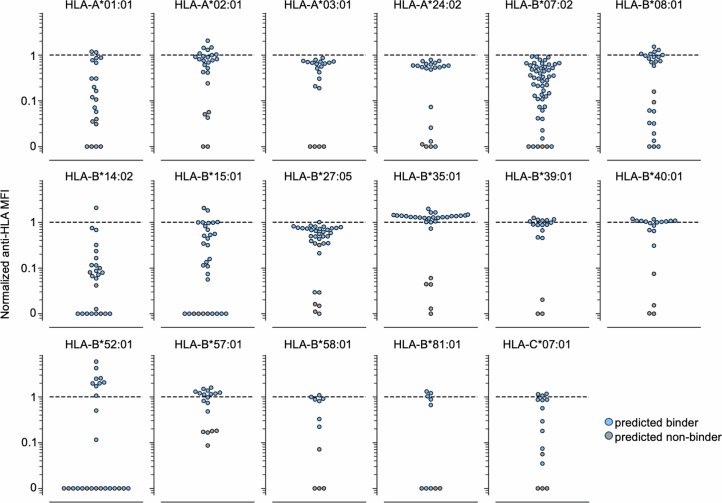
HLA class I-peptide stability assay data of predicted binder and non-binder peptides for each allele. MFI is normalized to the MFI of the known HIV immunodominant peptide (dashed line) for each allele. Blue circles indicate predicted binders and gray circles indicate predicted non-binders.

**Extended Data Fig. 5:**
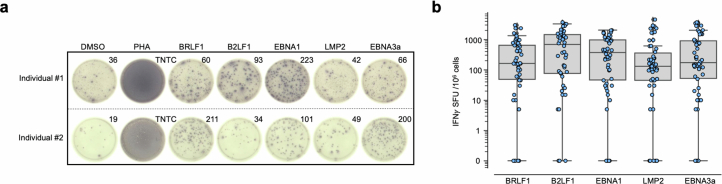
Response of individuals in cohort to overlapping peptide pools from Epstein-Barr Virus (EBV) proteins. **(a)** Representative IFN-*γ* ELISpot assays for representative HLA-haplotyped individuals in cohort using overlapping peptide pools from EBV proteins BRLF1, B2LF1, EBNA1, LMP2 and EBNA3a. DMSO was used as a negative control and phytohemagglutinin-L (PHA) was used as a positive control. TNTC: Too Numerous To Count. **(b)** Summary data for ELISpot data for all individuals in cohort (n = 50 peptides per protein). Box plots are presented with medians as center lines, 25^th^ and 75^th^ percentiles as lower and upper quartiles, and 1.5 x interquartile range from the quartiles as whiskers (outliers not shown).

For each HLA-I allele, we evaluated the HLA-I-binding and stabilization capacity of 9–52 predicted peptide binders and 3–5 predicted non-binders. When normalized to the HLA-I stabilizing mean fluorescence intensity (MFI) of a corresponding immunodominant HLA-I-restricted HIV epitope^37^, the predicted binders have a significantly higher MFI than predicted non-binders (Fig. 5c–e and Extended Data Figs. 4 and 5). For the selected top-337 ranked peptides used in the HLA-I–peptide stability assay, the median MUNIS score for a predicted binder was 0.96, with 318 HLA-I–peptide pairs scoring ≥0.50, 285 pairs ≥0.70, 219 pairs ≥0.90 and 187 pairs ≥0.95. This provided the opportunity to assess the relationship between quantitative MUNIS score and experimental HLA-I–peptide stabilization, revealing that higher MUNIS score thresholds for classifying peptides led to increased discrimination between binders and non-binders (Fig. 5f).

## MUNIS identifies established and novel immunogenic EBV epitopes

**Fig. 6:**
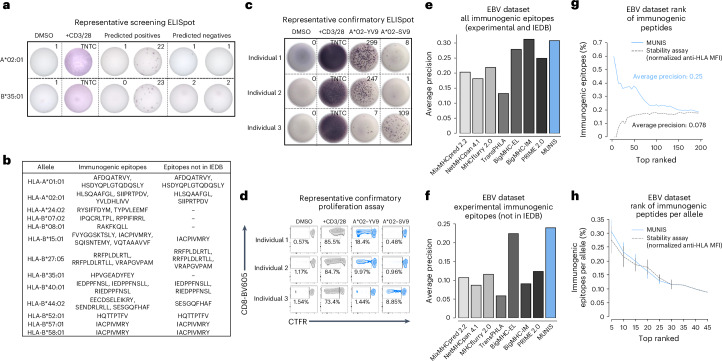
MUNIS identifies established and novel EBV CD8^+^ T cell epitopes. **a**, Representative IFNγ ELISpot assays for HLA-A*02:01- and HLA-B*35:01-restricted peptides from the EBV proteome within individuals who express these HLA-I alleles. DMSO was used as a negative control and soluble CD3 and CD28 antibodies were used as a positive control. TNTC, too numerous to count. All peptides were tested as technical duplicates. **b**, List of all 25 unique immunogenic epitopes from EBV identified by IFNγ ELISpot as well as 12 epitopes not currently deposited in the IEDB as HLA binders or immunogenic peptides. **c**, IFNγ ELISpot assays for immunogenic HLA-A*02:01 peptides from the EBV proteome for three individuals who are HLA-A*02:01^+^. DMSO was used as a negative control and soluble CD3 and CD28 antibodies were used as a positive control. **d**, Proliferation of CD8^+^ T cells from the 3 individuals above when stimulated for 5 days with an immunogenic HLA-A*02:01 epitope. All peptides were tested as technical duplicates. **e**,**f**, Average precision of MUNIS and current state-of-the-art tools MixMHCpred 2.2, NetMHCpan 4.1, MHCflurry 2.0, TransPHLA, BigMHC and PRIME in predicting all experimentally determined immunogenic epitopes and those derived from the IEDB (8–14mers) (**e**) or solely restricted to those novel epitopes that were experimentally confirmed (**f**). **g**,**h**, Percentage of experimentally determined and known immunogenic EBV epitopes in the top-*n* ranked tested peptides as predicted by MUNIS or the HLA-I stability assay (**g**) and further stratified by HLA-I allele with no restriction on epitope length (8–15mers) (**h**). *N* = 15, 14, 12, 8, 4, 2, 1, 1 and 1 alleles with sufficient data to calculate the fraction of immunogenic epitopes in the top-5, -10, -15, -20, -25, -30, -35, -40 and -45 ranked peptides per allele, respectively. Data are presented as mean values ± standard error of the mean. Four of the immunogenic epitopes were excluded from the analyses in **g** and **h** given the absence of corresponding monoallelic TAP-deficient HLA-I cell lines and HLA-I–peptide stability measurements.

To assess the ability of MUNIS to identify immunogenic epitopes from EBV proteins (BRLF1, B2LF1, EBNA1, LMP2 and EBNA3a), we performed IFNγ ELISpot assays on PBMCs from 30 HLA-I-typed individuals using overlapping 15mer peptide pools from each protein. This demonstrated that all individuals had non-zero IFNγ ELISpot responses to at least one of the five overlapping EBV peptide pools (Extended Data Fig. 5), providing strong rationale for testing individual predicted peptides within these individuals. We therefore assessed T cell reactivity using an IFNγ ELISpot by matching individual peptides to participants with the requisite restricting HLA-I allele (Fig. 6a). Given the HLA-I haplotypes of our cohort, we expanded the set of HLA-I–peptide pairs to 370 to include HLA-A*11:01-, B*44:02- and C*08:02-peptide pairs, which were not present in HLA-I–peptide stability assessments. Of these 370 unique HLA-I–peptide pairs tested, we identified 27 HLA-I–peptide pairs and 25 unique peptides that elicited detectable T cell responses. Interestingly, 12 immunogenic peptides predicted by our model had not previously been identified or deposited in the IEDB (Fig. 6b), illustrating the ability of MUNIS to predict novel CD8^+^ T cell epitopes, even for an extensively studied pathogen such as EBV. Using ex vivo IFNγ ELISpot and proliferation assays, we further confirmed that one of the novel HLA-A*02:01-restricted EBV epitopes (SIIPRTPDV, BZLF1: 229–237) as well as a MUNIS-predicted, known immunodominant EBV epitope (YVLDHLIVV, BRLF1: 109–117) are capable of eliciting both effector (Fig. 6c) and memory (Fig. 6d) CD8^+^ T cell responses across multiple individuals, illustrating the ability of MUNIS to identify broadly reactive epitopes.

Because our cohort was limited to 30 individuals, we supplemented our list of IFNγ ELISpot-confirmed immunogenic epitopes with CD8^+^ T cell reactivity data from the IEDB. Thus, in addition to the 27 pairs found to be immunogenic, we included any peptide with a positive frequency of response reported in the IEDB via an established T cell assay (for example, tetramer staining, IFNγ ELISpot or intracellular cytokine staining). This revealed an additional 18 HLA-I–peptide pairs, for a total of 45 pairs with 42 unique peptides. Compared with other computational epitope prediction algorithms, MUNIS showed enhanced identification of immunogenic EBV epitopes. MUNIS ranked four immunogenic epitopes in the top 5 and 20 in the top-60 ranked peptides. When compared with other tools, MUNIS and BigMHC-IM perform best, with an average precision of 0.3 (Fig. 6e). In terms of novel immunogenic epitopes (that is, those not currently in the IEDB), MUNIS outperforms all other tools (Fig. 6f). While these comparisons are performed on epitopes with lengths of 8–14 residues (the peptide length training range for several tools), one of the highly ranked immunogenic peptides for HLA-A*02:01 is a 15mer (HSDYQPLGTQDQSLY, LMP2A: 71-85), illustrating the ability of MUNIS to predict longer epitopes. Notably, without restriction on peptide length, MUNIS outperformed the HLA-I–peptide stability assay in immunogenicity prediction (Fig. 6g) and had comparable results when stratified by HLA-I allele (Fig. 6h). Collectively, these results confirm that MUNIS can identify not only binders but also immunogenic epitopes from a de novo pathogen with high accuracy.

## Discussion

In this study, we report the development of MUNIS, a deep learning model for CD8^+^ T cell epitope prediction within foreign pathogens. We utilized a deep learning architecture and curated dataset for HLA-I epitope presentation and implemented the encoding of HLA-I–peptide sequences with a protein language model, utilized as a deep transformer model^24^. These models are trained on millions of protein sequences and learn features that are broadly applicable to property-prediction tasks^24^, making them well suited for accurate immunogenic epitope prediction. Similar to MHCflurry2.0^14^, we used context from the parent protein to capture epitope processing. This composite of characteristics led to improved performance in the prediction of HLA-I-presented peptides with increased probability of being targeted by multiple individuals.

Despite notable improvements in predicting epitope presentation, we did not observe substantial discrimination between immunogenic and non-immunogenic epitopes when conditioned on peptides presented by HLA-I in influenza immunopeptidomic datasets. This may be due to the ability of MUNIS to enhance prediction of peptide presentation itself and not necessarily predict immunogenicity of filtered HLA-I binders. Similarly, this may explain its improved performance ranking immunogenic epitopes in EBV when seen as a de novo virus, where all aspects of the model could be leveraged. In addition, we also found that MUNIS showed noticeable improvement compared with contemporary tools in predicting immunodominance hierarchies in acute and chronic HIV infection. Immunodominance hierarchies can be partially dictated by the dependence of HLA-I alleles on the protein tapasin^42^ by aligning hierarchical responses with HLA-I–peptide stability^43^. Because MUNIS ranks immunogenic epitopes comparably to experimental HLA-I–peptide stability, this may explain its increased efficacy in recapitulating immunodominance hierarchies. Notably, we find that BigMHC (when using both its eluted ligand EL and immunogenicity IM models) is the only predictor comparable to MUNIS across different evaluation settings. However, MUNIS performs consistently on all metrics using a single output score, making it broadly useful for presentation and immunodominance prediction.

An extensive ablation analysis revealed that the pretrained ESM-2 model and incorporation of flanking residues contribute to the outperformance of MUNIS on HLA-I–peptide prediction. As the model pretrained on 8 million parameters performed similarly to the one pretrained on 35 million parameters, we opted for the smaller model to increase efficiency. Given that the prediction task focuses on a single protein family, the benefits of deconvoluting underlying structural features across the full protein landscape by a larger language model may be lesser. Using percentage rank and the native score of the model to predict HLA-I–peptide binding partners performed similarly on immunodominance datasets, highlighting that MUNIS has learned to compare scores across HLA-I alleles despite inherent differences in sample sizes across alleles within the training data. Finally, ablation analysis on the decoy sampling method revealed similar results whether decoys were derived from Swiss-Prot or the human proteome. Overall, this analysis showed that while modelling features selectively benefit presentation prediction, some subtly detract from immunogenicity prediction. Thus, we offer versions of MUNIS trained with and without flanking residues.

Importantly, we note limitations to this work. First, the training and evaluation data of 205 prominent HLA-I alleles only partially captures the extent of HLA polymorphism in the population. In addition, while MUNIS captures features of immunogenic peptides, it does not substantially improve immunogenicity prediction when peptides are filtered on HLA-I binding. This would benefit from conditioning predictions based on the likelihood of HLA-I–peptide complexes engaging specific T cell receptors. However, the lack of available data^29,44–46^ for T cell receptor–peptide–HLA-I binding makes this task challenging. We also note that our model is largely trained on mass spectrometry data, which may contain potential biases such as under-representation of cysteine residues^47^. In addition, while we find that our architecture decisions result in improved presentation prediction, some features resulted in weaker immunogenicity prediction. This suggests that the transfer between the two tasks, although positive, is not completely linear.

Nonetheless, the ability of MUNIS to accurately predict immunogenic peptides and identify new epitopes has several implications. Rationally designing immunogens that will elicit robust T cell responses is pivotal to vaccine design, and MUNIS could therefore greatly accelerate this process. Surprisingly, we found that MUNIS outperformed an experimental HLA-I–peptide stability assay in predicting immunogenic epitopes. Thus, it may be possible to substantially reduce the experimental burden that accompanies binding and stability assays and directly perform immunogenicity studies on MUNIS-predicted peptides. Lastly, MUNIS was able to identify several novel CD8^+^ T cell epitopes in EBV, highlighting the potential of deep learning for epitope discovery. We envision that future efforts with expanded training datasets of immunogenic peptides will further improve model performance.

## Methods

### Datasets

The eluted ligand (that is, presentation) training set consists of four different datasets: the eluted ligand data deposited in the IEDB^20^ as well as data from the NetMHCpan4.1^13^, MHCflurry2.0^14^ and MixMHCpred2.2^16^ studies. The final dataset after filtering out epitopes in the evaluation dataset is composed of 651,237 positive peptide–HLA-I pairs and 3,701,209 negative decoys with peptide lengths between 8 and 15. Five-amino-acid-long N- and C-terminal flanking sequences for each peptide were fetched from the parent protein sequence annotated in the IEDB. When not available, we attempted to find a matching sequence by searching the Swiss-Prot sequences using MMSeqs2^48^. The dataset was filtered to data points where the parent protein could be identified. Sequences for the HLA alleles were obtained from the IMGT database^49^. Contrary to previous work that uses pseudo-sequences, that is, sequences of HLA molecules that are within peptide-binding range^50^, our model takes as input the full α1 and α2 domains of the HLA sequence, specifically the 180 residues ranging from positions 27 to 207.

### Model architecture

The binding module uses the ESM-2 protein language model of 6 million parameters^51^. Language models are trained on millions of protein sequences and have been shown to implicitly learn various structural features of proteins from sequence alone. The language model takes as input the HLA and peptide sequences in the form: [CLS] HLA [SEP] PEPTIDE [EOS], where the [CLS], [SEP] and [EOS] tokens indicate the start, separator and end of the sequence, respectively. After encoding the sequence with the language model, we use the representation of the [CLS] token as sequence representation, which is then fed to a two-layer feed-forward network. The loss is the binary cross entropy between the output scores and the ground-truth labels. As opposed to using only the output embeddings from the language model as features, we fine-tune the full ESM-2 language model during training.

The processing module uses a bidirectional long short-term memory (LSTM) recurrent neural network^52^, which is fed the peptide sequence as one-hot encoded amino acids, including its left and right flanks, corresponding to five amino acids on the N- and C-terminal ends of the peptide in its parent protein of origin. To allow the network to determine which of the amino acids belong to the peptide sequence and not to the flanks, we include a binary feature at each amino acid position. The output of the LSTM is a sequence of vectors that we pool into a single vector representation for the sequence by averaging along the sequence length dimension. Finally, we concatenate this feature vector with one from the binding module and feed it into a two-layer feed-forward network to produce the presentation score.

### Model training

We train our models using the PyTorch framework on 4× A6000 graphic processing units for 75,000 steps with a total batch size of 256 (2 hours of total training time). The binding model is initialized using the pretrained ESM-2 8 million parameter model and all the weights are fine-tuned during training. The LSTM used to model flanking residues is randomly initialized at the start of training. Our models are trained using half mixed-precision (fp16) using the Adam optimizer, with a learning rate of 1 × 10^−4^ that is constant throughout training.

### Peptide synthesis reagents

Fmoc-protected amino acids and synthesis resin, 2-chlorotrityl chloride, were purchased from Akaal Organics. Dimethylformamide (DMF), *N*-methyl pyrrolidone (NMP), acetonitrile and methyl *tert*-butyl ether (MTBE) were purchased from Fisher Bioreagents. 2-(6-Chloro-1-H-benzotriazole-1-yl)-1,1,3,3-tetramethylaminium hexafluorophosphate (HCTU) was purchased from AAPPTEC. Piperidine and dichloromethane (DCM) were from EMD-Millipore. Diisopropylethylamine (DIEA), *N*-methyl-morpholine (NMM), triisoprpopyl-silane, 3,6-dioxa-1,8-octanedithiol (DODT) and trifluoroacetic acid (TFA) were purchased from Sigma-Aldrich.

### Peptide synthesis and analysis

Peptides were synthesized on an automated robotic peptide synthesizer (AAPPTEC, Model 396 Omega) by using Fmoc solid-phase chemistry^53^ on 2-chlorotrityl chloride resin^54^. The C-terminal amino acids were loaded using the respective Fmoc amino acids in the presence of DIEA. Unreacted sites on the resin were blocked using methanol, DIEA and DCM (15:5:80 v/v). Subsequent amino acids were coupled using optimized (to generate peptides containing more than 90% of the desired full-length peptides) cycles consisting of Fmoc removal (deprotection) with 25% piperidine in NMP followed by coupling of Fmoc amino acids using HCTU/NMM activation. Each deprotection or coupling was followed by several washes of the resin with DMF to remove excess reagents. After the peptides were assembled and the final Fmoc group removed, peptide resin was then washed with DMF, DCM and methanol three times each and air-dried. Peptides were cleaved from the solid support and deprotected using odour-free cocktail (TFA/triisopropyl silane/water/DODT; 94/2.5/2.5/1.0 v/v) for 2.5 h at room temperature^55^. Peptides were precipitated using cold MTBE. The precipitate was washed 2 times in MTBE, dissolved in a solvent (0.1% TFA in 30% acetonitrile/70% water) followed by freeze-drying. Peptides were characterized by ultra-performance liquid chromatography and matrix assisted laser desorption/ionization mass spectrometry. All peptides were dissolved initially in 100% DMSO at a concentration of 40 mM, before dilution at the appropriate concentration in RPMI-1640 medium.

### HLA-I–peptide stability assay

HLA-I–peptide stability assays were performed as previously described^27^. In brief, 5 × 10^4^ Transporter Associated with Antigen Processing (TAP)-deficient monoallelic HLA-I-expressing 721.221 cells were incubated with 100 μM of peptide and 3 μg ml^−1^ of β2-microglobulin (Sino Biological) in RPMI-1640 medium overnight at 26 °C/5% CO_2_ for 18 h. Controls without peptide but with DMSO were performed in parallel. Following overnight incubation, cells were incubated at 37 °C/5% CO_2_ for 2 h before staining with live/dead violet viability dye (Life Technologies) and pan-HLA-ABC-APC antibody (Clone W6/32, BioLegend, 1:100 dilution). HLA-I surface expression was analysed by flow cytometry.

### Calculation of Shannon entropy

Shannon entropy (that is, sequence conservation scores) at each HLA anchor residue position was calculated using the following formula:

$$S=-{\sum }_{i}^{n}P\left({x}_{i}\right)\log_{2}P\left({x}_{i}\right),$$

where *n* is the number of unique amino acids at any given position, *x*_*i*_ is the *i*th unique amino acid, and *P*(*x*_*i*_) is the probability of amino acid *x*_*i*_ at that given position, calculated by dividing the number of peptides with amino acid *x*_*i*_ at the position of interest by the total number of peptides in the dataset.

### Study participants

Study participants were recruited from outpatient clinics at local Boston area clinics and from outside Boston. The Institutional Review Board of Massachusetts General Hospital approved the studies of cells derived from human blood samples. PBMCs from HIV^+^ individuals with viral loads below 2,000 copies either on or off suppressive anti-retroviral therapy were collected by Ficoll gradient separation from acid citrate dextrose tubes or leukapheresis samples. They were then cryopreserved and stored in liquid nitrogen for future use. High-resolution HLA-I typing was performed for all patients as described previously^56^. In brief, locus-specific PCR primers were used to amplify polymorphic exons of *HLA-A*, *HLA-B* and *HLA-C* genes with the Fluidigm Access Array (Fluidigm). PCR amplicons were pooled and sequenced on an Illumina MiSeq platform (Illumina). HLA alleles and genotypes were called using the Omixon HLA Explore (beta version) software (Omixon). Ambiguous calls were resolved by Sanger sequencing.

### Ex vivo IFNγ ELISpot assay

IFNγ ELISpot assays were performed according to the manufacturer’s instructions (Mabtech). PBMCs were incubated with individual peptides from EBV at a final concentration of 0.5 μg ml^−1^ for 16–18 h. Positive controls were anti-human CD3 (Clone OKT3, BioLegend, 0.5 μg ml^−1^) and anti-human CD28 (Clone CD28.2, BioLegend, 0.5 μg ml^−1^) antibodies. The number of spot-forming units in the highest-value DMSO control well was subtracted from each experimental well. Responses were considered positive if both replicates had greater than or equal to five spot-forming units per well above background.

### Ex vivo proliferation assay

PBMCs were suspended at 1 × 10^6^ cells per ml in 1 μM CellTrace Far Red dye (ThermoFisher) in PBS and incubated at 37 °C for 20 min. Cells were protected from light and mixed every 5 min during the incubation. RPMI supplemented with 10% fetal bovine serum was added to quench the reaction for 5 min, followed by centrifugation at 1,500 rpm for 3 min before resuspension in culture media. Cells were plated into 96-well U-bottom plates (Corning) (200,000 cells per well in 200 μl of culture media) and incubated with individual EBV peptides at a concentration of 0.5 μg ml^−1^ for 5 days. Cells were washed with PBS supplemented with 2% fetal bovine serum and stained with anti-human CD3-BUV395 (Clone UCHT1, BD Biosciences, 1:100 dilution), anti-human CD4-PE-Cy7 (Clone OKT4, BioLegend, 1:100 dilution), anti-human CD8-BV605 (Clone SK1, BioLegend, 1:100 dilution) and live/dead violet viability dye (Life Technologies). Cells were washed and fixed in 2% paraformaldehyde and flow cytometric analysis was performed on a BD LSR Fortessa (BD Biosciences).

### Reporting summary

Further information on research design is available in the Nature Portfolio Reporting Summary linked to this article.

## Supplementary information

Reporting Summary

## Figures and Tables

**Extended Data Table 1:**
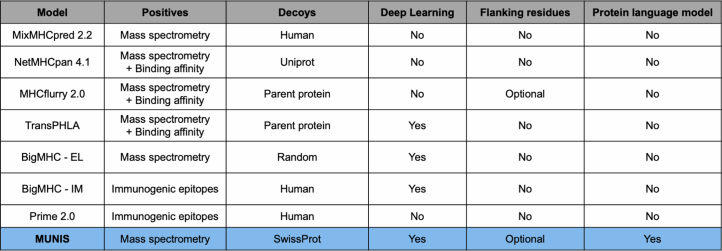
Features of MUNIS and competing predictive models

Table showing the differences between MUNIS and other models. Models in the literature differ based on several factors, including the sampling of negatives, model architecture and the use of flanking residues.

**Extended Data Table 2:**
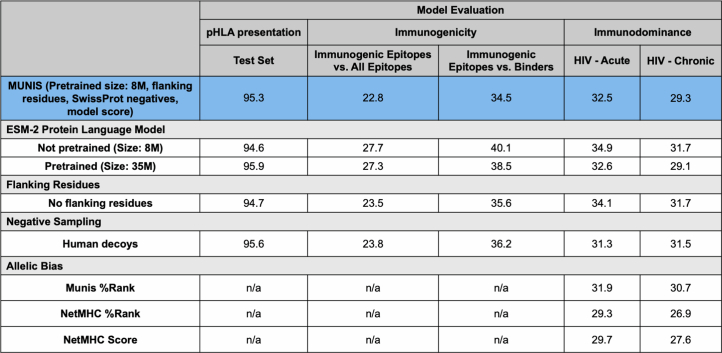
Ablation analysis of MUNIS deconvolutes features contributing to enhanced prediction of HLA-I-peptide binders and immunogenic epitopes compared to competing models

Ablation analysis showing the performance of MUNIS as a function of various modifications. All numbers are provided as median average precision stratified per allele, except for HIV immunodominance which is given as spearman coefficient. All ablated models were trained as an ensemble of five models to match the original MUNIS training schema.

**Extended Data Table 3:**
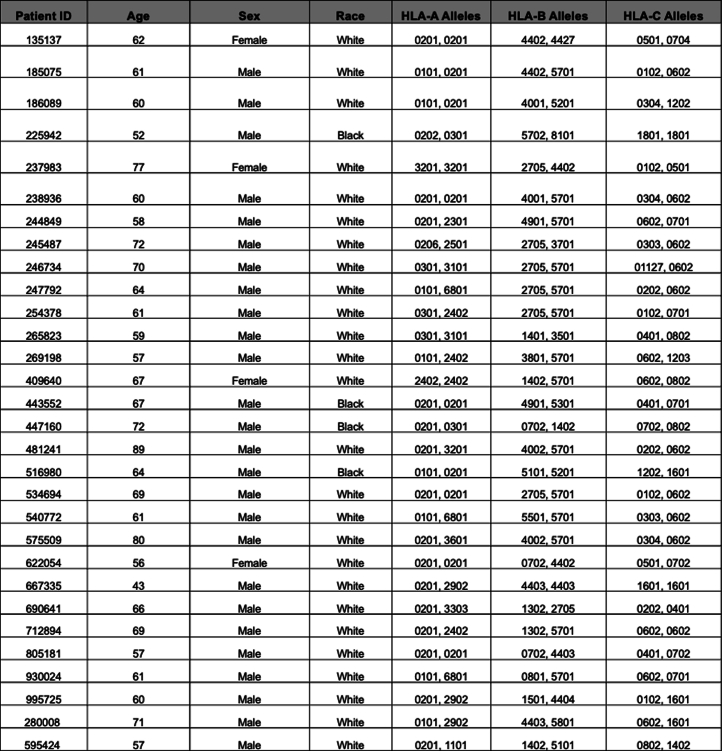
Characteristics of patient cohort utilized for assessment of immunogenicity of EBV peptides

N/A

## Data Availability

All data required to train and evaluate the models, HLA-I–peptide stability assay and ELISpot data are deposited in the Mendeley Data repository^57^. Sequences for the major histocompatibility complex alleles were obtained from the IMGT^49^ and can also be found alongside the released code. Full viral protein sequences, including accession codes, are also available in the Mendeley Data repository.

## Code availability

The code used to train the model and run predictions using our trained model weights is available via Zenodo at 10.5281/zenodo.14219509 (ref. ^58^).
