# Supplementary material for: Deep learning enhances the prediction of HLA class I-presented CD8+ T cell epitopes in foreign pathogens
Source: Nat Mach Intell. 2025 Jan 28;7(2):232–43. doi: 10.1038/s42256-024-00971-y (PMC11847706; doi:10.1038/s42256-024-00971-y)
Supplement: Supplementary file 1 — Reporting Summary [file 42256_2024_971_MOESM1_ESM.pdf]

nature portfolio

Corresponding author(s): Gaurav Gaiha

Last updated by author(s): November 26, 2024

Reporting Summary

Nature Portfolio wishes to improve the reproducibility of the work that we publish. This form provides structure for consistency and transparency in reporting. For further information on Nature Portfolio policies, see our Editorial Policies and the Editorial Policy Checklist.

Statistics

For all statistical analyses, confirm that the following items are present in the figure legend, table legend, main text, or Methods section.

n/a	Confirmed	
☐	☒	The exact sample size (*n*) for each experimental group/condition, given as a discrete number and unit of measurement
☐	☒	A statement on whether measurements were taken from distinct samples or whether the same sample was measured repeatedly
☐	☒	The statistical test(s) used AND whether they are one- or two-sided<br>*Only common tests should be described solely by name; describe more complex techniques in the Methods section.*
☐	☒	A description of all covariates tested
☒	☐	A description of any assumptions or corrections, such as tests of normality and adjustment for multiple comparisons
☐	☒	A full description of the statistical parameters including central tendency (e.g. means) or other basic estimates (e.g. regression coefficient) AND variation (e.g. standard deviation) or associated estimates of uncertainty (e.g. confidence intervals)
☐	☒	For null hypothesis testing, the test statistic (e.g. F, t, r) with confidence intervals, effect sizes, degrees of freedom and P value noted<br>*Give P values as exact values whenever suitable.*
☒	☐	For Bayesian analysis, information on the choice of priors and Markov chain Monte Carlo settings
☒	☐	For hierarchical and complex designs, identification of the appropriate level for tests and full reporting of outcomes
☐	☒	Estimates of effect sizes (e.g. Cohen's *d*, Pearson's *r*), indicating how they were calculated

Our web collection on statistics for biologists contains articles on many of the points above.

Software and code

Policy information about availability of computer code

Data collection	No software was used for data collection.
Data analysis	Data analysis was performed using standard python packages (pandas, matplotlib). Model training was done using machine learning frameworks (pytorch, pytorch-lightning).

For manuscripts utilizing custom algorithms or software that are central to the research but not yet described in published literature, software must be made available to editors and reviewers. We strongly encourage code deposition in a community repository (e.g. GitHub). See the Nature Portfolio guidelines for submitting code & software for further information.

Data

Policy information about availability of data

All manuscripts must include a data availability statement. This statement should provide the following information, where applicable:

- Accession codes, unique identifiers, or web links for publicly available datasets
- A description of any restrictions on data availability
- For clinical datasets or third party data, please ensure that the statement adheres to our policy

All data required to train and evaluate the models and raw stability assay and ELISpot data are deposited in the Mendeley Data repository with DOI: 10.17632/5w2zg5jn27.1. Dataset used for training were extracted from the publicly available NetMHCpan4.1 (https://doi.org/10.1093/nar/gkaa379), MHCflurry2.0 (https://doi.org/10.1016/j.cels.2020.06.010), and MixMHCpred2.2 (https://doi.org/10.1016/j.cels.2022.12.002) studies. Evaluation data was taken from the IEDB

(https://www.iedb.org/), Calis et al (https://doi.org/10.1371/journal.pcbi.1003266), and Pyke et al (https://doi.org/10.1016/j.mcpro.2021.100111). Sequences for the MHC alleles were obtained from the IMGT (https://www.ebi.ac.uk/ipd/imgt/hla/) and can also be found alongside the released code. Full viral protein sequences, including accession codes, are also available in the Mendeley Data repository.

Human research participants

Policy information about studies involving human research participants and Sex and Gender in Research.

Reporting on sex and gender	We have reported sex and race in Extended Table 3 of the manuscript.
Population characteristics	Population characteristics are reported in Extended Table 3 of the manuscript.
Recruitment	Study participants included those that had been recruited by the Ragon Institute of Mass General, MIT and Harvard. HLA typing had previous been obtained on all participants. Sex and/or gender of participants was determined based on self-report.
Ethics oversight	All study participants provided written informed consent. The study was approved by the Mass General Brigham Institutional Review Board.

Note that full information on the approval of the study protocol must also be provided in the manuscript.

Field-specific reporting

Please select the one below that is the best fit for your research. If you are not sure, read the appropriate sections before making your selection.

☒ Life sciences ☐ Behavioural & social sciences ☐ Ecological, evolutionary & environmental sciences

For a reference copy of the document with all sections, see nature.com/documents/nr-reporting-summary-flat.pdf

Life sciences study design

All studies must disclose on these points even when the disclosure is negative.

Sample size	A total of 30 HLA-typed participants were selected for analysis. Written informed consent was obtained from all participants.<br><br>Sample size was not calculated due to the extensive degree of HLA polymorphism in the human population. We therefore selected individuals with HLA class I alleles that matched those that were present in our HLA class I-peptide stability assessments and computational predictions.
Data exclusions	No data were excluded.
Replication	All experiments were performed in technical duplicates and all attempts at replication were successful.
Randomization	No randomization was performed as participants were assigned to epitope immunogenicity assessments based on their HLA class I haplotype.
Blinding	No blinding was performed and sample names were labeled based on their de-identified ID, sample collection date and time points. No blinding was done to avoid sample cross-contamination.

Reporting for specific materials, systems and methods

We require information from authors about some types of materials, experimental systems and methods used in many studies. Here, indicate whether each material, system or method listed is relevant to your study. If you are not sure if a list item applies to your research, read the appropriate section before selecting a response.

Materials & experimental systems

n/a	Involved in the study	
☐	☒	Antibodies
☐	☒	Eukaryotic cell lines
☒	☐	Palaeontology and archaeology
☒	☐	Animals and other organisms
☒	☐	Clinical data
☒	☐	Dual use research of concern

Methods

n/a	Involved in the study	
☒	☐	ChIP-seq
☐	☒	Flow cytometry
☒	☐	MRI-based neuroimaging

Antibodies

Antibodies used	Anti-human CD3 (Clone OKT3, BioLegend, Cat # 317302, Lot B358930), anti-human CD28 (Clone CD28.2, BioLegend, Cat # 302902, Lot B394362), anti-human CD3-BUV395 (Clone UCHT1, BD Biosciences, Cat # 563546, Lot 3072678), anti-human CD4-PE-Cy7 (Clone OKT4, BioLegend, Cat # 317414, Lot B357837), anti-human CD8-BV605 (Clone SK1, BioLegend, Cat # 344742, Lot B370756), pan-HLA-ABC-APC antibody (Clone W6/32, BioLegend, Cat # 311410, Lot B373456).
Validation	Antibodies validated by manufacturers and confirmed to bind populations of interest.

Eukaryotic cell lines

Policy information about cell lines and Sex and Gender in Research

Cell line source(s)	All cell lines used were derived from human female B cell line 721.221 (https://doi.org/10.4049/jimmunol.142.9.3320). HLA-monoallelic TAP-deficient cell lines were developed previously using CRISPR-Cas9 technology (https://doi.org/10.1016/j.celrep.2021.109378).
Authentication	Each monoallelic TAP-deficient cell lines was authenticated using surface staining for HLA expression and CRISPR amplicon sequencing to confirm TAP1 gene editing (https://doi.org/10.1016/j.celrep.2021.109378).
Mycoplasma contamination	Cell lines are routinely tested for mycoplasma contamination.
Commonly misidentified lines (See ICLAC register)	N/A

Flow Cytometry

Plots

Confirm that:

☒ The axis labels state the marker and fluorochrome used (e.g. CD4-FITC).

☒ The axis scales are clearly visible. Include numbers along axes only for bottom left plot of group (a 'group' is an analysis of identical markers).

☒ All plots are contour plots with outliers or pseudocolor plots.

☒ A numerical value for number of cells or percentage (with statistics) is provided.

Methodology

Sample preparation	Details in Methods section. Patient PBMCs were extracted from whole blood using Ficoll gradient separation.
Instrument	BD LSR II (H47200037)
Software	BD FACSDiva Software was used to collect the data and FlowJo was used to analyze the data.
Cell population abundance	No cell sorting was performed in this paper.
Gating strategy	Gating strategy for HLA class I-peptide stability assays: FSC-A/SSC-A --> SSC-H/SSC-W --> Live/Dead Violet/SSC-A --> HLA-APC histogram. Gating strategy for proliferation assays: FSC-A/SSC-A --> SSC-H/SSC-W --> CD3-BUV395/Live/Dead Violet --> CD8-BV605/CD4-PECy7 --> CellTrace Far Red (CTFR)/CD8-BV605.

☒ Tick this box to confirm that a figure exemplifying the gating strategy is provided in the Supplementary Information.
